# Tunable Structural Color in Au-Based One-Dimensional Hyperbolic Metamaterials

**DOI:** 10.3390/nano15241898

**Published:** 2025-12-17

**Authors:** Ricardo Téllez-Limón, René I. Rodríguez-Beltrán, Fernando López-Rayón, Mauricio Gómez-Robles, Katie Figueroa-Guardiola, Jesús E. Chávez-Padua, Victor Coello, Rafael Salas-Montiel

**Affiliations:** 1 SECIHTI—Centro de Investigación Científica y de Educación Superior de Ensenada, Unidad Académica Monterrey, Alianza Centro 504, PIIT, Apodaca 66629, Nuevo León, Mexico; rrodrigu@cicese.mx; 2 Tecnologico de Monterrey, Escuela de Ingeniería y Ciencias, Eugenio Garza Sada 2501 Sur, Col: Tecnológico, Monterrey 64700, Nuevo León, Mexico; fernando.rayon@tec.mx (F.L.-R.);; 3 Laboratory Light, Nanomaterials, and Nanotechnology—L2n CNRS UMR 7076, Université de Technologie de Troyes, 10004 Troyes, France; mauricio.gomez_robles@utt.fr (M.G.-R.); rafael.salas_montiel@utt.fr (R.S.-M.); 4 EUT+ Institute of Nanomaterials and Nanotechnologies—EUTINN, European University of Technology, European Union, 10004 Troyes, France; 5 Centro de Investigación Científica y de Educación Superior de Ensenada, Unidad Académica Monterrey, Alianza Centro 504, PIIT, Apodaca 66629, Nuevo León, Mexico; vcoello@cicese.mx

**Keywords:** metaphotonics, structural color, nano-optics, metamaterials

## Abstract

Structural coloration arising from nanoscale light–matter interactions has emerged as a key research area in nanophotonics. Among the various materials investigated, noble metals—particularly gold—play a central role due to their well-defined plasmonic response and chemical stability, but their structural coloring typically requires complex and highly engineered nanostructures. However, modern photonic technologies demand scalable approaches to produce structural colors that can be finely tuned. In this contribution, we experimentally and numerically demonstrate the fine tunability of structural color in gold-based one-dimensional hyperbolic metamaterials (1D-HMMs) by varying their structural parameters: number of layers (*N*), period (*T*), and filling fraction (*p*). Our results show that variations in *N* lead to changes in luminance with minimal shifts in chromaticity, while variations in *T* introduce moderate color shifts without affecting luminance. In contrast, changes in *p* produce the largest modifications in chromaticity, though the trend is non-monotonic and less predictable. These findings highlight the potential of 1D-HMMs for achieving finely controlled gold-based coloration for advanced photonic technologies.

## 1. Introduction

Color is one of the most immediate and powerful visual features in nature. Beyond its aesthetic appeal, it plays a fundamental role in communication, camouflage, signaling, and functionality across biological systems [1,2,3]. In general, when illuminating an object, certain wavelengths of incident light are absorbed by pigments or dyes, while the remaining wavelengths are reflected or transmitted in a process known as subtractive coloration [4,5]. However, when the object contains subwavelength features, it can scatter light in specific directions, leading to constructive or destructive interference between waves. In combination with subtractive effects, this interference gives rise to structural colors, whose appearance depends on viewing angle, illumination conditions, and eye perception [6,7,8]. Many of the most striking natural colors are not produced by pigments or dyes but result from structural coloration, such as those observed in butterfly wings, bird feathers, or beetle shells [9,10,11]. Understanding how these structural colors are formed is not only essential for unraveling biological complexity but also for enabling cutting-edge technologies such as advanced displays [12,13,14,15], anti-counterfeiting [16,17,18,19], optical sensors [20,21], and high-efficiency photonic devices [22,23,24,25].

In recent years, advances in nanotechnology have opened new frontiers for designing and controlling structural colors in artificial materials. By precisely engineering light–matter interactions at the nanoscale, it is now possible to achieve vivid, durable, and tunable colors that go far beyond the capabilities of traditional pigments [26,27,28,29,30,31,32,33,34,35,36]. This progress has been enabled by the development of metamaterials: artificially structured composites that derive their optical properties not only from their composition but also from their geometry, making them especially suited for controlling the propagation of light [37,38,39,40,41].

In this context, gold (Au) is one of the most widely used materials in nanophotonics and holds particular interest not only for its historical and aesthetic value, but also for its remarkable optical properties, chemical stability, and durability at the nanoscale [42,43,44]. These characteristics have been widely exploited in plasmonics for the development of diverse nanophotonic technologies, including biosensors [45,46,47,48], optical filters [49,50,51], structured light [52,53], quantum platforms [54,55,56], and structural color generation [28,31,57,58]. However, structural coloration using Au has traditionally relied on metasurfaces with resonant nanostructures of complex geometries, such as plasmonic nanoantennas [13,28,31,59]. Although these designs can produce vivid and diverse colors, they often result in discrete or abrupt spectral features, making it difficult to finely and continuously control the perceived color. Overcoming this limitation is crucial for advancing technologies that require precise spectral engineering or tunable appearance across a well-defined range, motivating the development of alternative design strategies.

Among the various types of metamaterials, one-dimensional hyperbolic metamaterials (1D-HMMs) stand out for their extreme anisotropy, high effective refractive index, and strong electromagnetic field confinement [60,61,62]. These structures consist of periodic arrays of alternating thin dielectric and metallic layers, forming a uniaxially anisotropic medium that exhibits hyperbolic dispersion [51,63]. This unique property enables unusual light–matter interactions, including an enhanced density of optical states, subwavelength confinement, directional propagation of light, and tunable absorption bands [51,60,61,63,64]. In addition to their rich optical behavior, 1D-HMMs offer significant practical advantages: they are straightforward to fabricate using conventional thin-film deposition techniques and allow for precise control over geometry through parameters such as period, layer thickness, number of layers, and material choice [51,63]. Despite extensive research into their plasmonic and dispersion properties, the potential of these multilayered structures for structural color generation and tunability remains largely unexplored [65,66].

Owing to their geometric flexibility and optical anisotropy, in this work we experimentally and numerically demonstrate that 1D-HMMs based on gold/titanium dioxide (Au/TiO_2_) offer an ideal platform for achieving fine control over structural color. By systematically varying the number of layers, period, and filling fraction of these minimalistic metamaterials, it is possible to achieve smooth control over the color appearance of Au within a warm color palette, ranging in the red-orange tones.

Numerical simulations based on the transmittance matrix method [51,67] and experimental measurements show that number of layers impact on the luminosity of samples with a minimal effect on color difference between samples, while period has an impact on the color difference without significantly modifying their luminosity. In contrast, variation in filling fraction simultaneously affects both luminosity and color, inducing a more complex and less predictable color tuning. These findings demonstrate the potential of 1D-HMMs as a versatile and practical platform for finely tuning the optical properties of metals like gold. Relying on standard thin-film deposition techniques, our approach enables a scalable and lithography-free route to structural color generation, offering new tools for applications that demand engineered appearance and precise spectral control.

## 2. Materials and Methods

### 2.1. One-Dimensional Hyperbolic Metamaterials

Hyperbolic metamaterials are subwavelength-structured media that exhibit anisotropic effective permittivity leading to hyperbolic dispersion relations. Consider harmonic electromagnetic plane waves, with fields described by $E=E_{0}e^{-i(\omega t-k\cdot r)}$ and $H=H_{0}e^{-i(\omega t-k\cdot r)}$, incident on an anisotropic medium characterized by the diagonal permittivity tensor

(1) $$\varepsilon =\left(\begin{array}{ccc} \varepsilon_{xx} & 0 & 0\\ 0 & \varepsilon_{yy} & 0\\ 0 & 0 & \varepsilon_{zz} \end{array}\right),$$

whith wavevector $k=(k_{x},k_{y},k_{z})$. Using Maxwell equations, it is possible to show that the wave equation in anisotropic media can be written as follows [60,62]:

(2) $$k\times \left(k\times E\right)+\omega^{2}\mu_{0}\varepsilon_{0}\varepsilon E=0.$$

Assuming a uniaxial medium with $\varepsilon_{xx}=\varepsilon_{yy}\equiv \varepsilon_{⊥}$ and defining $k_{⊥}^{2}=k_{x}^{2}+k_{y}^{2}$, the non-trivial solution of the vector wave equation yields the dispersion relation [60]

(3) $$\left(k_{⊥}^{2}+k_{z}^{2}-\varepsilon_{⊥}k_{0}^{2}\right)\left(\frac{k_{⊥}^{2}}{\varepsilon_{zz}}+\frac{k_{z}^{2}}{\varepsilon_{⊥}}-k_{0}^{2}\right)=0.$$

In Equation (3), the first factor describes a spheroid surface corresponding to an ordinary (isotropic-like) wave. The second factor defines an ellipsoid. However, if we consider extreme anisotropy, that is, opposite signs of the real part of $\varepsilon_{⊥}$ and $\varepsilon_{zz}$, the Equation (3) describes a hyperbolic surface in the k-space. Materials satisfying this condition are referred to as one-dimensional hyperbolic metamaterials (1D-HMMs). Because metals feature a negative real permittivity at optical frequencies, 1D-HMMs can be realized by periodically alternating dielectric and metallic thin layers [50,51]. It is important to clarify that 1D-HMMs are distinct from one-dimensional metallic nanoparticles, whose applications involve energy transfer or enhanced adsorption [68,69].

1D-HMMs are classified according to the sign of the real part of the components of the permittivity tensor. If $\varepsilon_{⊥}>0$ and $\varepsilon_{zz}<0$, the 1D-HMM is known as dielectric hyperbolic or HMM type I. If $\varepsilon_{⊥}<0$ and $\varepsilon_{zz}>0$, it is named metallic hyperbolic or type II. If $\varepsilon_{⊥}>0$ and $\varepsilon_{zz}>0$ the metamaterial is an effective dielectric, and if $\varepsilon_{⊥}<0$ and $\varepsilon_{zz}<0$ the metamaterial is an effective metal. For subwavelength multilayers, the sign of the components $\varepsilon_{⊥}$ and $\varepsilon_{zz}$ can be derived from the effective medium theory [50,62]

(4) $$\varepsilon_{⊥}=p\;\varepsilon_{m}+\left(1-p\right)\;\varepsilon_{d},$$

(5) $$\varepsilon_{zz}=\frac{p}{\varepsilon_{m}}+\frac{1-p}{\varepsilon_{d}},$$

where *p* is the filling fraction (portion of metal in a period), and $\varepsilon_{m}$, $\varepsilon_{d}$ are the dielectric functions of metal and dielectric layers, respectively.

### 2.2. Fabrication of the Samples

**Figure 1:**
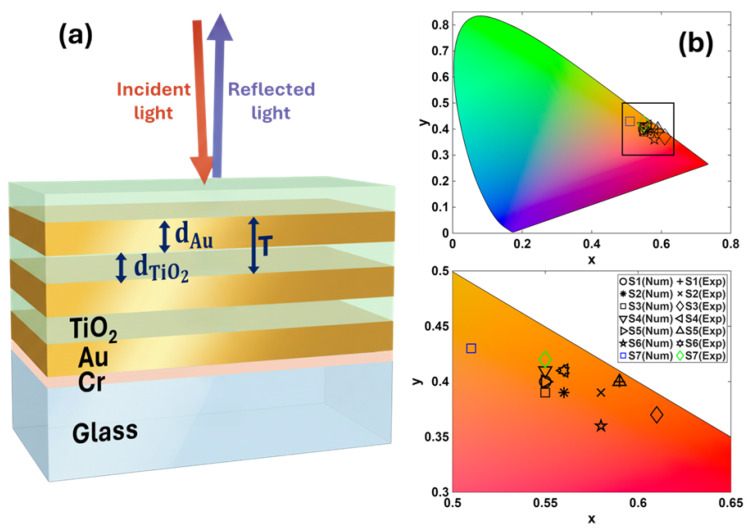
Structural colors with 1D-HMMs. (**a**) Schematic representation of the periodic multilayered structure consisting of a glass substrate on top of which was deposited a 3 nm Cr adhesive layer, and then Au and TiO_2_ thin layers of thickness $d_{Au}$ and $d_{TiO_{2}}$. Incident and reflected light spectra were measured from the air superstrate. (**b**) Chromaticity diagram of the colors obtained for different 1D-HMMs studied in this work. All colors remain in the red-orange region with warm tones.

The one-dimensional hyperbolic metamaterials studied in this work consisted of periodically alternating thin films of gold (Au) and titanium dioxide (TiO_2_). These multilayers were deposited on glass coverslips with a refractive index $n_{sub}=1.51$, as shown in Figure 1. Au layers were deposited by Joule heating, while TiO_2_ layers were deposited using electron beam evaporation. Both depositions were performed sequentially in the same chamber (MEB4000, PLASSYS, Marolles-en-Hurepoix, France) without breaking the vacuum. Prior to deposition, the coverslips were cleaned in an ultrasonic bath with isopropyl alcohol and deionized water. The chamber was evacuated to a base pressure of $5\times 10^{-7}$ Torr. Deposition was carried out at a pressure of $5\times 10^{-6}$ Torr at a constant rate of 1 Å/s. To ensure adhesion of the first Au thin layer, a 3 nm chromium (Cr) adhesion layer was deposited onto the glass substrate.

### 2.3. Numerical Simulations

The reflection spectra of the multilayered hyperbolic metamaterials were numerically computed using the transmittance matrix method [51,67]. In these simulations, both the glass substrate and the air superstrate were modeled as semi-infinite media. The reflectance was calculated as the average response to TE- and TM-polarized plane wave illumination. The geometrical parameters of the multilayered structure included the number of layers (*N*) and the thicknesses of Au ($d_{Au}$) and TiO_2_ ($d_{TiO_{2}}$) thin films. The period of the structure was defined as $T=d_{Au}+d_{TiO_{2}}$, while the filling fraction was given by the portion of Au within a single period, $p=d_{Au}/T$. The electric permittivity of Au was calculated using the Drude-Lorentz model, as described in references [70,71]. The permittivity values for TiO_2_ and Cr were taken from references [72] and [73], respectively. Constant refractive indices were assumed for the glass substrate ($n_{sub}=1.51$) and the air superstrate ($n_{sup}=1.0$).

### 2.4. Experimental Setup

**Figure 2:**
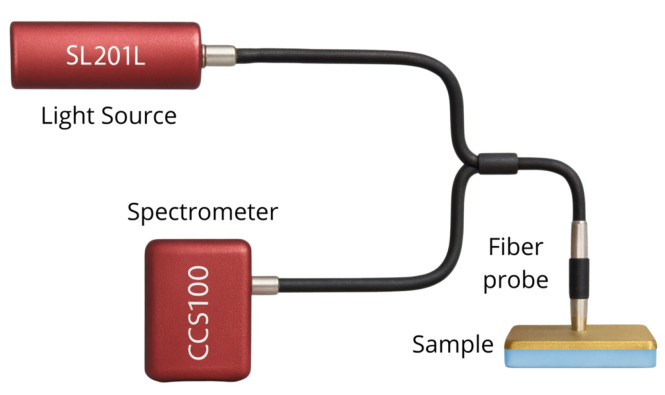
Schematic representation of the colorimeter used for specular detection of reflected power from the fabricated samples. The fiber probe was placed at 3 mm above the surface of the sample. The XYZ color coordinates were obtained according to CIE 1931 standard considering the light spectrum of the Tungsten-Halogen lamp.

The color of the samples was measured using a colorimeter with specular detection of the reflected light. This experimental characterization was performed using a Tungsten-Halogen light source (SLS201L, Thorlabs, Newton, NJ, USA) as illuminant, coupled to a multicore reflection probe fiber bundle (RP22, Thorlabs, Newton, NJ, USA) to illuminate the sample. The second port of the fiber bundle was connected to a spectrometer (CCS100, Thorlabs, Newton, NJ, USA). The fiber probe was positioned at approximately 3 mm above the sample surface and oriented perpendicularly to collect the power of light reflected by the sample, as depicted in Figure 2. All measurements were performed in a darkened environment to minimize ambient light contamination [74]. The integration time for the spectrometer was set to 100 ms, and 1000 reflection spectra were averaged for each measurement, including background spectra for correction. Measurements were repeated in four different regions of each sample to ensure consistency, and the average results were used. Color analysis was performed based on the CIE 1931 standard [75,76,77], as described in Section 2.5, covering a wavelength range from 380 nm to 740 nm.

### 2.5. Color Determination

Color measurements in this study were based on the CIE 1931 standard colorimetric system [75,76]. This color space provides a linear representation of color perception based on human vision, where all visible colors are defined by positive *X*, *Y*, and *Z* values. The *X* component is related to red-green sensitivity, *Y* corresponds to brightness with peak sensitivity in the green region, and *Z* is mainly associated with blue light sensitivity.

The tristimulus values, *X*, *Y*, and *Z*, were computed over the spectral wavelength range from 380 nm to 740 nm, according to the equations [75,76]:

(6) $$X=K\sum_{\lambda =380}^{740}S\left(\lambda \right)R\left(\lambda \right)\bar{x}\left(\lambda \right)\;\Delta \lambda =K\sum_{\lambda =380}^{740}P_{r}\left(\lambda \right)\bar{x}\left(\lambda \right)\;\Delta \lambda ,$$

(7) $$Y=K\sum_{\lambda =380}^{740}S\left(\lambda \right)R\left(\lambda \right)\bar{y}\left(\lambda \right)\;\Delta \lambda =K\sum_{\lambda =380}^{740}P_{r}\left(\lambda \right)\bar{y}\left(\lambda \right)\;\Delta \lambda ,$$

(8) $$Z=K\sum_{\lambda =380}^{740}S\left(\lambda \right)R\left(\lambda \right)\bar{z}\left(\lambda \right)\;\Delta \lambda =K\sum_{\lambda =380}^{740}P_{r}\left(\lambda \right)\bar{z}\left(\lambda \right)\;\Delta \lambda ,$$

with the normalization factor

(9) $$K=\frac{100}{\sum_{\lambda =380}^{740}S\left(\lambda \right)\bar{y}\left(\lambda \right)\;\Delta \lambda }.$$

In these equations, S(λ) is the spectral power distribution of the illuminant, R(λ) is the spectral reflectance of the sample, and Δλ is the wavelength step. The functions $\bar{x}\left(\lambda \right)$, $\bar{y}\left(\lambda \right)$, and $\bar{z}\left(\lambda \right)$ are the CIE 1931 color matching functions. Since the reflected power $P_{r}\left(\lambda \right)$ results from the modulation of the reflectance of the sample R(λ) by the illuminant spectrum S(λ), we directly used $P_{r}\left(\lambda \right)=S\left(\lambda \right)R\left(\lambda \right)$ to compute the experimental XYZ coordinates.

To graphically represent color coordinates, we used the CIE 1931 chromaticity diagram, which displays all chromaticities visible to human eye, regardless of brightness. This bidimensional diagram, with coordinates (x,y), is enclosed in a horseshoe-shaped boundary, where the edge corresponds to pure spectral (monochromatic) colors, and points inside represent their mixtures (hue and saturation). The chromaticity coordinates are given by [75,76]:

(10) $$x=\frac{X}{X+Y+Z},$$

(11) $$y=\frac{Y}{X+Y+Z}.$$

To quantify perceptual color differences, the computed XYZ values were converted to the CIELAB color space. This color space is based on human eye perceptual uniformity and is defined through three coordinates $\left(L^{*},a^{*},b^{*}\right)$. $L^{*}$ tells how light or dark the colors appear ($L^{*}=0$ means black and $L^{*}=100$ means white). The coordinate $a^{*}$ tells if the color is more reddish or greener (large $-a^{*}$ means more green, large $+a^{*}$ means redder). The coordinate $b^{*}$ tells if the color is more yellowish or bluish (large $-b^{*}$ means more blue, large $+b^{*}$ means more yellow). The CIELAB components $\left(L^{*},a^{*},b^{*}\right)$ were computed according to [75,77]:

(12) $$L^{*}=116\cdot f\left(\frac{Y}{Y_{N}}\right)-16,$$

(13) $$a^{*}=500\cdot \left[f\left(\frac{X}{X_{N}}\right)-f\left(\frac{Y}{Y_{N}}\right)\right],$$

(14) $$b^{*}=200\cdot \left[f\left(\frac{Y}{Y_{N}}\right)-f\left(\frac{Z}{Z_{N}}\right)\right],$$

where the function f(α) is defined as follows:

(15) $$f\left(\alpha \right)=\left\{\begin{array}{cc} \alpha^{1/3}, & \mathrm{if}\;\alpha >\delta^{3}\\ \frac{\alpha }{3\delta^{2}}+\frac{4}{29}, & \mathrm{if}\;\alpha \leq \delta^{3} \end{array}\right.,$$

being δ=6/29 the nonlinear correction factor for visual perception. The reference white tristimulus values of the Tungsten-Halogen lamp were calculated as $X_{N}=K_{\mathrm{lamp}}\cdot \sum_{\lambda =380}^{740}S_{\mathrm{lamp}}\left(\lambda \right)\;\bar{x}\left(\lambda \right)\;\Delta \lambda =108.8865$, $Y_{N}=K_{\mathrm{lamp}}\cdot \sum_{\lambda =380}^{740}S_{\mathrm{lamp}}\left(\lambda \right)\;\bar{y}\left(\lambda \right)\;\Delta \lambda =100.00$, and $Z_{N}=K_{\mathrm{lamp}}\cdot \sum_{\lambda =380}^{740}S_{\mathrm{lamp}}\left(\lambda \right)\;\bar{z}\left(\lambda \right)\;\Delta \lambda =21.7622$. The normalization constant for the lamp is given by $K_{\mathrm{lamp}}=100/\sum_{\lambda =380}^{740}S_{\mathrm{lamp}}\left(\lambda \right)\;\bar{y}\left(\lambda \right)\;\Delta \lambda$, being $S_{lamp}\left(\lambda \right)$ its spectral power distribution, while $\bar{x}\left(\lambda \right)$, $\bar{y}\left(\lambda \right)$, and $\bar{z}\left(\lambda \right)$ are the color matching functions of the CIE 1931 XYZ color space.

The CIELAB color space allows us to determine the chroma, denoted as $C^{*}=\sqrt{{\left(a^{*}\right)}^{2}+{\left(b^{*}\right)}^{2}}$, which quantifies color vividness or saturation, or simply, how colorful it appears compared to a gray of the same lightness. The difference in chroma between two samples, $\Delta C^{*}$, tells us variations in saturation, independently of lightness. Additionally, this color space allows us to quantify the perceptual difference between two colors using the metric ΔE, which is defined as the Euclidean distance between two points in the CIELAB color space:

(16) $$\Delta E=\sqrt{{\left(L_{2}^{*}-L_{1}^{*}\right)}^{2}+{\left(a_{2}^{*}-a_{1}^{*}\right)}^{2}+{\left(b_{2}^{*}-b_{1}^{*}\right)}^{2}}.$$

To enable digital visualization of the resulting colors, the XYZ tristimulus coordinates were converted to linear RGB values using the standard transformation matrix for the sRGB color space [76,77]:

(17) $$\left[\begin{array}{c} R\\ G\\ B \end{array}\right]=\left[\begin{array}{ccc} 3.2406 & -1.5372 & -0.4986\\ -0.9689 & 1.8758 & 0.0415\\ 0.0557 & -0.2040 & 1.0570 \end{array}\right]\cdot \left[\begin{array}{c} X\\ Y\\ Z \end{array}\right].$$

To reproduce perceptually accurate and display-ready colors, each linear RGB value was gamma-corrected according to the sRGB standard [76,77]:

(18) $$\gamma_{\mathrm{sRGB}}\left(\beta \right)=\left\{\begin{array}{cc} 12.92\cdot \beta , & \beta \leq 0.0031308\\ 1.055\cdot \beta^{\frac{1}{2.4}}-0.055, & \beta >0.0031308 \end{array}\right.,$$

with β∈R,G,B. All RGB values were clipped in the [0,1] range to ensure compatibility with standard display devices and to eliminate numerical artifacts from the transformation.

It is worth mentioning that, at the final stage of manuscript preparation, ChatGPT (OpenAI, GPT-4) was used exclusively for grammar checking and language refinement, without influencing the scientific results. The authors reviewed and verified all content, taking full responsibility for the final manuscript.

## 3. Results

### 3.1. Single Au Thin Layer

To assess how the color of a single Au thin layer changes when incorporated into a multilayered structure with dielectric spacers (1D-HMM), we first measured the color of a 50 nm Au layer deposited on a glass cover-slip, with a 3 nm Cr adhesion layer.

**Figure 3:**
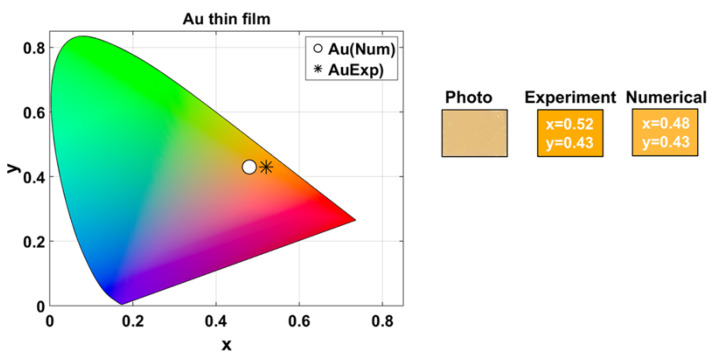
Color of a 50 nm Au layer on a glass substrate. The CIE 1931 chromaticity coordinates (x,y) and RGB images illustrate the characteristic golden appearance of the sample.

Figure 3 shows the location of the (x,y) chromaticity coordinates in the CIE 1931 diagram, alongside a photograph of the surface of the fabricated sample, taken with a smartphone camera (Samsung Galaxy A54) under laboratory illumination. The Figure also displays the RGB colors derived from both experimental and numerical data, revealing the characteristic golden appearance of the thin layer of Au.

The experimental tristimulus values were $X_{Au,exp}=67.95$, $Y_{Au,exp}=56.81$, and $Z_{Au,exp}=6.20$, while the numerical simulated values were $X_{Au,num}=65.81$, $Y_{Au,num}=59.82$, and $Z_{Au,num}=12.66$. Based on the equations provided in Section 2.5, these values correspond to the CIELAB coordinates $L_{Au,exp}^{*}=80.07$, $a_{Au,exp}^{*}=13.15$, and $b_{Au,exp}^{*}=34.03$ for the experimental data and $L_{Au,num}^{*}=81.74$, $a_{Au,num}^{*}=1.43$, and $b_{Au,num}^{*}=1.54$ for the numerical results. The corresponding chroma values are $C_{Au,exp}^{*}=36.48$ and $C_{Au,num}^{*}=2.10$, resulting in a chroma difference $\Delta C^{*}=34.38$. The total color difference was $\Delta E_{Au}=34.57$.

The close agreement in lightness and the pronounced chroma difference indicate that the color discrepancy between the experimental and numerically simulated results primarily arises from chromaticity rather than brightness variations. This chroma difference mainly arises from surface scattering due to sub-wavelength imperfections, deviations of the actual thickness from the nominal values, and the material dispersion models used. Despite these discrepancies, the qualitative perceived color remains consistent (both appear orange-yellowish), allowing for meaningful comparison of the overall color evolution.

### 3.2. Multilayered One-Dimensional Hyperbolic Metamaterials

To study the influence of structural parameters on the color of one-dimensional hyperbolic metamaterials, we fabricated seven samples consisting of periodic arrays of Au-TiO_2_ thin layers. Three parameters were varied: the number of layers (*N*), the period (*T*), and the filling fraction (*p*). *N* indicates the total number of layers of the samples excluding the 3 nm Cr adhesion layer present in all samples. The period *T* is defined as the combined thickness of two adjacent Au and TiO_2_ layers, $T=d_{Au}+d_{TiO_{2}}$, where $d_{Au}$ and $d_{TiO_{2}}$ are the individual layer thicknesses. The filling fraction, *p*, is given by the portion of Au in each period, $p=d_{Au}/T$.

The structural parameters and the experimental and numerical values of the XYZ tristimulus coordinates for each sample are listed in Table 1. As noted, samples S1, S2, and S3 have the same period (T=50 nm) and the same filling fraction (p=0.5) but vary in the number of layers (N∈[6,8,10]). Samples S4, S1 and S5 share the same number of layers (N=6), the same filling fraction (p=0.5), and variable period (T∈[40,50,70] nm). The samples S6, S1 and S7 have the same number of layers (N=6), the same period (T=50 nm), but a variable filling fraction (p∈[0.4,0.5,0.8]).

#### 3.2.1. Classification of 1D-HHMs

**Figure 4:**
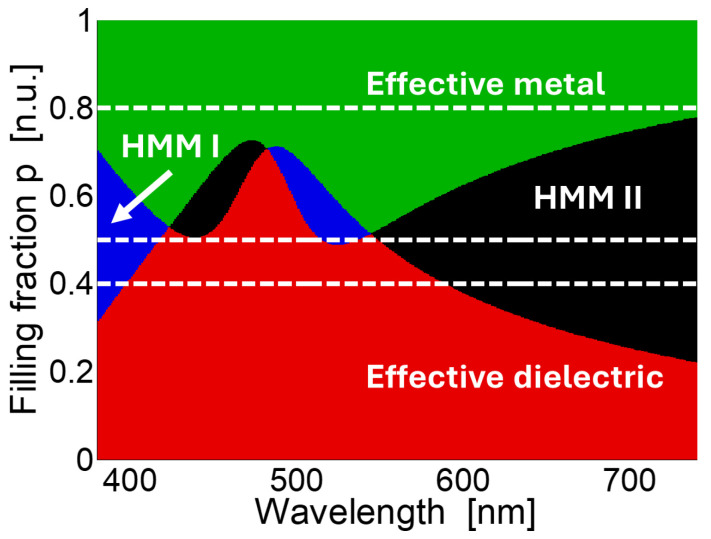
Effective medium classification of the Au/TiO_2_ based 1D-HMMs as a function of wavelength and filling fraction. Red denotes the effective dielectric regime, green the effective metallic regime, blue the type I hyperbolic regime, and black the type II hyperbolic regime. Horizontal dashed lines mark the filling fractions of the fabricated samples (p=[0.4,0.5,0.8]).

Figure 4 shows the effective-medium classification of the Au/TiO_2_ multilayered structure as a function of wavelength and filling fraction. The color map identifies four different optical regimes: effective metallic (green), effective dielectric (red), hyperbolic type I (blue), and hyperbolic type II (black). The horizontal dashed lines indicate filling fractions p=[0.4,0.5,0.8], corresponding to the nominal values of the fabricated samples.

As shown in Figure 4, multilayers with small filling fractions behave as effective dielectrics, where the reflected spectrum is dominated by photonic effects. For large filling fractions, the structure approaches a thick-metal limit, exhibiting mirror-like reflectance. In the intermediate range p≈0.3−0.7, the multilayers cross into the hyperbolic regimes, where the reflection arises from the interplay of photonic and plasmonic contributions.

#### 3.2.2. Effect of Variation in the Number of Layers

To study the effect of the variation in the number of layers in structural coloring, we compared samples S1, S2, and S3. The corresponding experimental and numerical CIELAB coordinates, $L^{*}a^{*}b^{*}$, and chroma, $C^{*}$, for these samples are given in Table 2. These values were computed according to the description given in Section 2.5. For each coordinate, the average value (mean) and the coefficient of variation (CV) are presented. Maximum chroma difference ($\Delta C_{max}^{*}$) and color difference ($\Delta E_{max}^{*}$) between two samples are also provided.

**Figure 5:**
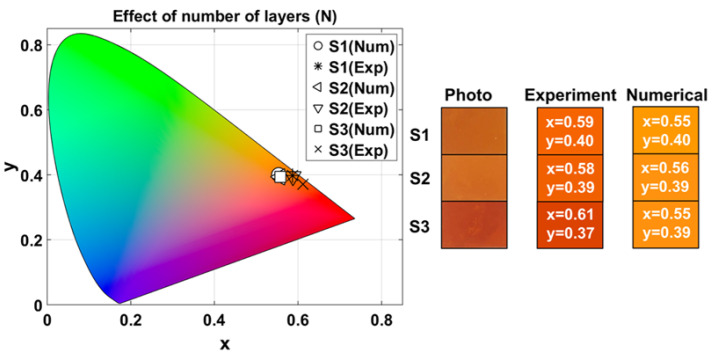
Experimental and numerical chromaticity coordinates of 1D-HMMs for different numbers of layers. Rectangles show the photograph of the surface of the fabricated samples under laboratory illumination, and RGB colors generated from experimental and numerical measurements obtained under illumination with Tungsten-Halogen lamp. The numbers inside the colored rectangles correspond to their respective (x,y) chromaticity coordinates.

Figure 5 shows the chromaticity diagram indicating the experimental and numerical chromaticity (x,y) coordinates of each sample. Experimentally, for S1, $\left(x_{exp},y_{exp}\right)=\left(0.59\pm 0.002,0.40\pm 0.002\right)$, while numerically $\left(x_{num},y_{num}\right)=\left(0.55,0.40\right)$. For S2, experimentally, $\left(x_{exp},y_{exp}\right)=\left(0.58\pm 0.0004,0.39\pm 0.0004\right)$, and numerically $\left(x_{num},y_{num}\right)=\left(0.56,0.39\right)$. For S3, $\left(x_{exp},y_{exp}\right)=\left(0.61\pm 0.0002,0.37\pm 0.0004\right)$, and $\left(x_{num},y_{num}\right)=\left(0.55,0.39\right)$. Figure 5 also presents a photograph of the surface of the fabricated samples under laboratory illumination, as well as the RGB colors generated from the experimental and numerical data obtained under illumination with the Tungsten-Halogen lamp.

Based on the average values and coefficients of variation (CV) of the coordinates $L^{*}$, $a^{*}$, and $b^{*}$ of Table 2, the samples are located within the warm red-orange region with medium-high luminosity, as observed in the color diagram of Figure 5. Both experimental measurements and numerical simulations follow the same general trend as the number of layers (*N*) increases: the reflectivity ($L^{*}$) decreases due to higher light absorption; the red-green chromaticity ($a^{*}$) remains in the red region with a slight increase; and the yellow-blue chromaticity ($b^{*}$) decreases but stays within the yellow range. As observed, chromaticity is relatively stable, whereas luminosity is more sensitive to changes in *N*.

The largest color difference ($\Delta E_{max}^{*}$) occurs between samples S1 and S3 in both the experimental and numerical results. This difference is mainly driven by variations in lightness ($L^{*}$) rather than chromaticity, because the maximum chroma difference ($\Delta C_{max}^{*}$) is comparatively small compared to $\Delta E_{max}^{*}$.

#### 3.2.3. Effect of Variation in the Period

We compared samples S4, S1, and S5 to analyze the effect of varying the period (T∈[40,50,70] nm) while keeping constant the number of layers (N=6) and the filling fraction (p=0.5). Table 3 lists the corresponding experimental and numerical CIELAB coordinates, $L^{*}a^{*}b^{*},$ and chroma, $C^{*}$, for these samples, as well as the mean values, coefficients of variation, maximum chroma difference ($\Delta C_{max}^{*}$) and maximum color difference ($\Delta E_{max}^{*}$) between samples.

**Figure 6:**
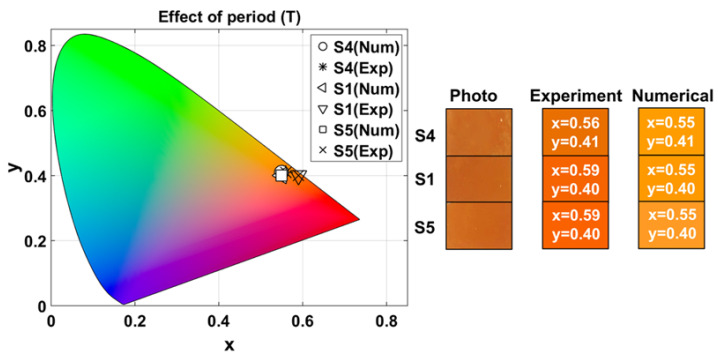
Experimental and numerical (x,y) chromatic coordinates as a function of period variation. Rectangles show the photograph of the surface of the fabricated samples under laboratory illumination, and RGB colors generated from experimental and numerical measurements obtained under illumination with the Tungsten-Halogen lamp. The numbers inside the colored rectangles correspond to their respective (x,y) chromaticity coordinates.

Figure 6 shows the experimental and numerical chromaticity (x,y) coordinates of each sample. Experimentally, for S4, $\left(x_{exp},y_{exp}\right)=\left(0.56\pm 0.0001,0.41\pm 0.0005\right)$, while numerically $\left(x_{num},y_{num}\right)=\left(0.55,0.41\right)$. For S1, experimentally $\left(x_{exp},y_{exp}\right)=\left(0.59\pm 0.002,0.40\pm 0.002\right)$, and numerically $\left(x_{num},y_{num}\right)=\left(0.55,0.40\right)$. For S5, $\left(x_{exp},y_{exp}\right)=\left(0.59\pm 0.001,0.40\pm 0.001\right)$, and $\left(x_{num},y_{num}\right)=\left(0.55,0.40\right)$. Photographs of the surface of the samples under laboratory illumination, and the RGB color generated from the corresponding experimental and numerical values of Table 3, obtained under illumination with the Tungsten-Halogen lamp, are also shown in Figure 6.

According to the $L^{*}$, $a^{*}$, and $b^{*}$ values listed in Table 3, all samples are located within the warm color region, particularly in the red-orange zone with medium-high lightness, as shown in Figure 6. As *T* increases, $L^{*}$ remains nearly constant, indicating that brightness is largely unaffected; $a^{*}$ shows a slight increase; and $b^{*}$ exhibits the most noticeable variation, though it stays within the yellow region.

#### 3.2.4. Effect of Variation in the Filling Fraction

To study the effect of the variation in the filling fraction on the structural coloring of our 1D-HMMs, we compared samples S6, S1, and S7. For these samples, both N=6 and T=50 nm were kept constant, while the filling fraction varied (p∈[0.4,0.5,0.8]). The corresponding experimental and numerical CIELAB coordinates, $L^{*}a^{*}b^{*}$, and chroma, $C^{*}$, for these samples, as well as the average (mean), coefficient of variation, maximum chroma difference ($\Delta C_{max}^{*}$), and maximum color difference ($\Delta E_{max}^{*}$) between samples, are given in Table 4.

The experimental chromaticity coordinates obtained for sample S6 were $\left(x_{exp},y_{exp}\right)=\left(0.56\pm 0.001,0.41\pm 0.001\right)$, and the numerical coordinates were $\left(x_{num},y_{num}\right)=\left(0.58,0.36\right)$. For S1, experimentally $\left(x_{exp},y_{exp}\right)=\left(0.59\pm 0.002,0.40\pm 0.002\right)$, and numerically $\left(x_{num},y_{num}\right)=\left(0.55,0.40\right)$. For S7, $\left(x_{exp},y_{exp}\right)=\left(0.55\pm 0.0002,0.42\pm 0.0001\right)$, and $\left(x_{num},y_{num}\right)=\left(0.51,0.43\right)$.

**Figure 7:**
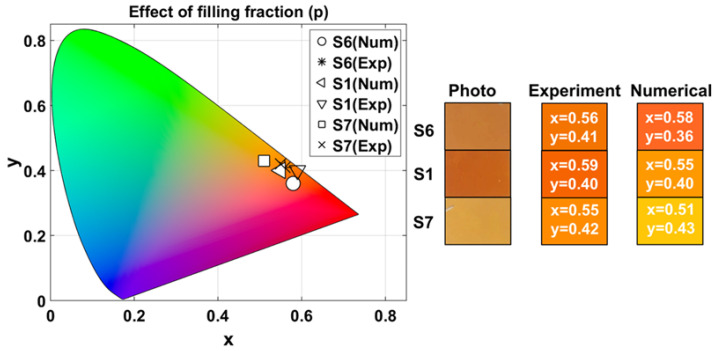
Chromatic diagram for experimental and numerical measurements of 1D-HMMs when varying filling fraction (*p*). Rectangles show photographs of the samples surface under laboratory illumination, and experimental and numerical RGB colors under Tungsten-Halogen lamp illumination. Numbers inside the rectangles correspond to their respective (x,y) chromaticity coordinates.

Figure 7 shows the chromaticity diagram with the experimental and numerical chromaticity coordinates (x,y) for each sample, photographs of the surface of the samples, and the RGB colors derived from the corresponding experimental and numerical values listed in Table 4.

The results in Table 4 show that all samples fall within the warm red-orange region with medium-high brightness, as illustrated in Figure 7. The maximum color difference is significantly larger compared to the variations in *N* and *T*. The color difference between samples is caused by a combined effect of changes in both luminance ($L^{*}$) and chromaticity ($a^{*},b^{*}$), unlike the previous cases where the variation was dominated by only one component.

This suggests that the filling fraction introduces a more complex and less predictable variation in perceived color. In other words, even though the variation in *p* allows for a wider color range, it offers less precise control over color tuning, since it modifies multiple perceptual components simultaneously. Therefore, if a specific chromaticity or luminance is desired, modifying *p* may require additional compensation strategies to maintain color accuracy.

## 4. Discussion

Experimentally, the measured chromaticities fall within $x_{exp}\in \left[0.5454,0.6114\right]$ and $y_{exp}\in \left[0.3713,0.4232\right]$, yielding spans $\Delta x_{exp}=0.066$ and $\Delta y_{exp}=0.0519$, with a maximum chromaticity displacement of $\Delta C_{exp}=30.53$. The numerical simulations show a comparable range, $x_{num}\in \left[0.5147,0.5825\right]$ and $y_{num}\in \left[0.3638,0.4285\right]$, corresponding to spans of $\Delta x_{num}=0.0678$ and $\Delta y_{num}=0.0647$, with a maximum chromaticity displacement of $\Delta C_{num}=36.98$. The experimentally measured minimum chroma and color differences were $\Delta C_{exp}=5.03$ and $\Delta E_{exp}^{*}=5.52$, whereas the numerical predictions yielded $\Delta C_{num}=0.12$ and $\Delta E_{num}^{*}=0.36$. The color of the samples is primarily confined to the red-orange spectral region due to the interband absorption of Au and the design of the multilayered structure, enabling fine control of structural color rather than broad spectral shifts.

In general, both experimental observations and numerical predictions indicate that increasing the number of layers *N* does not significantly affect the color hue, while the brightness is reduced. The chromaticity coordinates *x* and *y* exhibit extremely low variations, confirming that the number of layers has a limited impact on the perceived hue. However, other aspects of color perception respond more noticeably to an increase in *N*: brightness ($L^{*}$) decreases, resulting in darker samples; $a^{*}$ increases slightly, but the color remains within the red region, and $b^{*}$ decreases, indicating a loss of yellow saturation and a shift toward more muted tones.

The chromaticity coordinates (x,y) barely change with period. Unlike the variation in *N*, the brightness ($L^{*}$) remains nearly constant. The coordinate $a^{*}$ increases with *T*, reflecting a growing red component, while $b^{*}$ is significantly affected when *T* is modified. Although varying the period does not influence hue or brightness, it has a noticeable impact on the blue-yellow balance. This indicates that *T* contributes to the warming of the color tone.

The filling fraction (*p*) was the most influential parameter for tuning the color appearance of gold-based 1D-HMMs. Whereas the colors remain within the warm color region (spanning from reddish-orange to yellow tones), changes in chromaticity and brightness are more pronounced, making precise color control more challenging.

Overall, the observed effects were more pronounced in the experimental data than in simulations, which can be attributed to several factors. The fabrication technique (thermal evaporation) inherently introduces subwavelength surface roughness that induces scattering and alters the reflection spectra. In addition, deviations between the nominal and actual layer thicknesses inevitably arise during deposition, leading to discrepancies with the idealized structures assumed in the simulations. These fabrication-related limitations could be partially mitigated using alternative techniques such as atomic layer deposition. Finally, the theoretical models used for the dielectric constants of the materials do not fully capture their behavior in the thin-film regime. Despite these limitations, the qualitative optical behavior and the resulting color trends remain consistent with the predicted multilayer response.

As demonstrated, 1D-HMMs provide a lithography-free platform for fine color tuning through controlled variations in structural parameters (number of layers, period, and filling fraction). This represents a significant advantage over metasurfaces based on complex nanostructured geometries, which, although capable of spanning wider color gamuts [17,21,57], often exhibit chromaticity shifts with reported color differences in $\Delta E^{*}>5$ due to fabrication tolerances [13], resulting in abrupt color transitions and reduced reproducibility. This work demonstrates that 1D-HMMs constitute a complementary platform for fine color control, which can be combined with nanolithography fabrication techniques to enable new opportunities in advanced photonic color technologies.

## Figures and Tables

**Table 1:** Structural parameters and XYZ tristimulus values experimentally and numerically obtained, for the fabricated and simulated samples.

Sample	N	p	T	$d_{\mathrm{Au}}$	$d_{{\mathrm{TiO}}_{2}}$	$X_{\exp}$	$Y_{\exp}$	$Z_{\exp}$	$X_{\mathrm{num}}$	$Y_{\mathrm{num}}$	$Z_{\mathrm{num}}$
			(nm)	(nm)	(nm)						
S1	6	0.5	50	25	25	41.86	28.37	1.07	82.05	59.25	6.74
S2	8	0.5	50	25	25	40.19	26.96	1.21	80.04	56.48	7.10
S3	10	0.5	50	25	25	31.35	19.04	0.88	79.28	55.87	7.03
S4	6	0.5	40	20	20	38.80	28.53	2.25	81.37	59.78	5.69
S5	6	0.5	70	35	35	43.41	28.96	0.79	82.77	60.08	8.90
S6	6	0.4	50	20	30	43.21	31.61	2.23	65.67	41.01	6.05
S7	6	0.8	50	40	10	53.66	41.64	3.09	95.22	79.27	10.50

**Table 2:** Experimental and numerical CIELAB color coordinates of 1D-HMMs as a function of number of layers (*N*). Both period (T=50 nm) and filling fraction (p=0.5) were constant.

		Experimental				Numerical			
ID	N	$L^{*}$	$a^{*}$	$b^{*}$	$C^{*}$	$L^{*}$	$a^{*}$	$b^{*}$	$C^{*}$
S1	6	60.23	35.01	58.07	67.73	81.43	35.04	32.61	47.91
S2	8	58.94	35.66	52.74	63.64	79.88	37.94	27.60	46.89
S3	10	50.74	42.53	46.26	62.63	79.54	37.98	27.49	46.84
	Mean	56.64	37.73	52.36		80.29	36.99	29.24	
	CV (%)	9.09	11.04	11.30		1.25	4.57	9.99	
		$\Delta E_{\max}^{*}$ = 16.68		$\Delta C_{\max}^{*}$ = 5.10		$\Delta E_{\max}^{*}$ = 6.09		$\Delta C_{\max}^{*}$ = 1.07	

**Table 3:** Experimental and numerical CIELAB color coordinates of 1D-HMMs as a function of period (*T*), with constant number of layers (N=6) and filling fraction (p=0.5).

		Experimental				Numerical			
ID	T (nm)	$L^{*}$	$a^{*}$	$b^{*}$	$C^{*}$	$L^{*}$	$a^{*}$	$b^{*}$	$C^{*}$
S4	40	60.37	25.29	37.79	45.29	81.72	32.52	40.56	51.97
S1	50	60.23	35.01	58.07	67.73	81.43	35.04	32.62	47.91
S5	70	60.75	37.19	65.91	75.60	81.89	34.41	20.26	39.93
	Mean	60.45	32.50	53.93		81.68	33.99	31.15	
	CV (%)	0.45	19.50	26.91		0.28	3.85	32.83	
		$\Delta E_{\max}$ = 30.33		$\Delta C_{\max}^{*}$ = 30.31		$\Delta E_{\max}$ = 20.36		$\Delta C_{\max}^{*}$ = 12.04	

**Table 4:** Experimental and numerical CIELAB color coordinates for 1D-HMMs when varying filling fraction (*p*) for fixed number of layers (N=6) and period (T=50 nm).

		Experimental				Numerical			
ID	p	$L^{*}$	$a^{*}$	$b^{*}$	$C^{*}$	$L^{*}$	$a^{*}$	$b^{*}$	$C^{*}$
S6	0.4	63.03	26.82	42.58	50.36	70.19	50.96	18.04	53.06
S1	0.5	60.23	35.01	58.07	67.73	81.43	35.04	32.62	47.91
S7	0.8	70.63	21.55	45.01	50.00	91.36	15.40	28.20	32.10
	Mean	64.63	27.79	48.56		80.99	33.80	26.28	
	CV (%)	8.33	24.39	17.16		13.08	52.69	28.44	
		$\Delta E_{\max}$ = 21.45		$\Delta C_{\max}^{*}$ = 17.73		$\Delta E_{\max}$ = 42.61		$\Delta C_{\max}^{*}$ = 20.96	

## Data Availability

The data presented in this study are available on request from the corresponding author.

## Abbreviations

The following abbreviations are used in this manuscript:

1D-HMMs	One-dimensional hyperbolic metamaterials
N	Number of layers
T	Period
p	Filling fraction
Au	Gold
TiO_2_	Titanium dioxide
